# The ChinaMAP analytics of deep whole genome sequences in 10,588 individuals

**DOI:** 10.1038/s41422-020-0322-9

**Published:** 2020-04-30

**Authors:** Yanan Cao, Lin Li, Min Xu, Zhimin Feng, Xiaohui Sun, Jieli Lu, Yu Xu, Peina Du, Tiange Wang, Ruying Hu, Zhen Ye, Lixin Shi, Xulei Tang, Li Yan, Zhengnan Gao, Gang Chen, Yinfei Zhang, Lulu Chen, Guang Ning, Yufang Bi, Weiqing Wang, The ChinaMAP Consortium, Yifei Zhang, Yuhong Chen, Jianmin Liu, Jie Hong, Weiqiong Gu, Shu Wang, Hongyan Zhao, Xiuli Jiang, Aijing Shan, Qing Zhang, Wei Di, Qing Su, Xuefeng Yu, Guijun Qin, Qin Wan, Guixia Wang, Feixia Shen, Zuojie Luo, Yingfen Qin, Li Chen, Yanan Huo, Qiang Li, Chao Liu, Youmin Wang, Shengli Wu, Tao Yang, Huacong Deng, Jiajun Zhao, Yiming Mu, Limin Wang, Wenhua Zhao, Qiang Gao, Qiaoxiu Wang, Shengqing Wan, Fengyu Li, Xuanlin Huang, Xueyan Cheng, Peide Huang, Junmei Xu, Weining Hu, Guojia Zhang, Shizhi Luo, Chen Wang, Sha Liu, Hongyi Song, Yanhua Chen, Lishi Wang, Bing Zeng, Yu Liu, Siyu Wang, Jinbo Wu, Jia Guo, Rong Zhao, Lingyu Wu, Zewei Xiong, Mengyao Wang

**Affiliations:** 1 National Clinical Research Centre for Metabolic Diseases, State Key Laboratory of Medical Genomics, Shanghai Clinical Center for Endocrine and Metabolic Diseases, Shanghai Institute for Endocrine and Metabolic Diseases, Ruijin Hospital, Shanghai Jiao Tong University School of Medicine, Shanghai, 200025 China; 2 National Research Center for Translational Medicine, National Key Scientific Infrastructure for Translational Medicine (Shanghai), Shanghai Jiao Tong University, Shanghai, 200240 China; 3 Zhejiang Provincial Center for Disease Control and Prevention, Hangzhou, 310006 Zhejiang China; 4 Affiliated Hospital of Guiyang Medical College, Guiyang, 550004 Guizhou China; 5 The First Hospital of Lanzhou University, Lanzhou, 730000 Gansu China; 6 Sun Yat-sen Memorial Hospital, Sun Yat-sen University, Guangzhou, 510120 Guangdong China; 7 Dalian Municipal Central Hospital, Dalian, 116033 Liaoning China; 8 Fujian Provincial Hospital, Fujian Medical University, Fuzhou, 350001 Fujian China; 9 Central Hospital of Shanghai Jiading District, Shanghai, 201800 China; 10 Union Hospital, Tongji Medical College, Huazhong University of Science and Technology, Wuhan, 430022 Hubei China; 11 Xinhua Hospital Affiliated to Shanghai Jiao Tong University School of Medicine, Shanghai, China; 12 Tongji Hospital, Tongji Medical College, Huazhong University of Science and Technology, Wuhan, Hubei, China; 13 The First Affiliated Hospital of Zhengzhou University, Zhengzhou, Henan, China; 14 The Affiliated Hospital of Southwest Medical University, Luzhou, Sichuan, China; 15 The First Hospital of Jilin University, Changchun, Jilin, China; 16 The First Affiliated Hospital of Wenzhou Medical University, Wenzhou, Zhejiang, China; 17 The First Affiliated Hospital of Guangxi Medical University, Nanning, Guangxi, China; 18 Qilu Hospital of Shandong University, Jinan, Shandong, China; 19 Jiangxi Provincial People’s Hospital Affiliated to Nanchang University, Nanchang, Jiangxi, China; 20 The Second Affiliated Hospital of Harbin Medical University, Harbin, Heilongjiang, China; 21 Jiangsu Province Hospital on Integration of Chinese and Western Medicine, Nanjing, Jiangsu, China; 22 The First Affiliated Hospital of Anhui Medical University, Hefei, Anhui, China; 23 Karamay Municipal People’s Hospital, Karamay, Xinjiang, China; 24 The First Affiliated Hospital of Nanjing Medical University, Nanjing, Jiangsu, China; 25 The First Affiliated Hospital of Chongqing Medical University, Chongqing, China; 26 Shandong Provincial Hospital Affiliated to Shandong University, Jinan, Shandong, China; 27 Chinese People’s Liberation Army General Hospital, Beijing, China; 28 National Center for Chronic and Noncommunicable Disease Control and Prevention, Chinese Center for Disease Control and Prevention, Beijing, China; 29 National Institute for Nutrition and Health, Chinese Center for Disease Control and Prevention, Beijing, China

**Keywords:** Bioinformatics, Population genetics

## Abstract

Metabolic diseases are the most common and rapidly growing health issues worldwide. The massive population-based human genetics is crucial for the precise prevention and intervention of metabolic disorders. The China Metabolic Analytics Project (ChinaMAP) is based on cohort studies across diverse regions and ethnic groups with metabolic phenotypic data in China. Here, we describe the centralized analysis of the deep whole genome sequencing data and the genetic bases of metabolic traits in 10,588 individuals from the ChinaMAP. The frequency spectrum of variants, population structure, pathogenic variants and novel genomic characteristics were analyzed. The individual genetic evaluations of Mendelian diseases, nutrition and drug metabolism, and traits of blood glucose and BMI were integrated. Our study establishes a large-scale and deep resource for the genetics of East Asians and provides opportunities for novel genetic discoveries of metabolic characteristics and disorders.

## Introduction

Metabolic diseases are becoming a major growing public health challenge and causes of morbidity and mortality in the world. The most common and important metabolic diseases, type 2 diabetes and obesity, are comprised of different subtypes requiring specific diagnosis and treatments. Understanding the genetic architecture of metabolic traits is crucial for individual risk assessment, prevention, and treatment of metabolic diseases. Applying a comprehensive genetic analysis of massive cohorts can provide a systematic approach and effective strategy for the discovery of novel markers and targets. The variant spectrum of coding and non-coding regions from population genomics promotes a further understanding of the genetic basis of complex metabolic traits and diseases. The findings from the genome-wide association studies (GWAS) and population genome sequencing projects construct the knowledge of variants associated with metabolic traits.^1,2^

Large-scale reference datasets of population-specific genomics are fundamental for drug development and precision medicine of Mendelian and common diseases. Importantly, common metabolic traits and diseases are characterized by genetic heterogeneity in population groups.^1,3^ The populations in the Europe and USA have magnificent databases of human genomics and bioinformatics, including the UKbiobank,^4^ The Genome Aggregation Database (gnomAD),^5^ 1000 Genomes Project (1KGP),^6^ deCODE genetics,^7^ the UK10K project,^8^ the DiscovEHR^9^ and Trans-Omics for Precision Medicine (TOPMed) Program.^10^ Recently, two studies reported population genomic dataset from Chinese non-invasive prenatal testing and Singapore Chinese population.^11,12^ However, the low-depth sequencing data in these datasets limit the quantity of high-quality variants and accuracy of individual variants, especially rare variants. Considering the huge differences of genetic background and population characteristics between East Asians and Europeans,^13^ and the lack of high-depth Chinese cohort genomic study, representative database from Chinese cohorts is a critical part for the missing diversity.

Here, we describe the genomic dataset and analysis of 10,588 deep whole genome sequencing (WGS) data from The China Metabolic Analytics Project (ChinaMAP). The ChinaMAP was designed to comprehensively characterize the diverse genetic architectures of Chinese Han and major ethnic minorities across different geographical areas, and investigate their contribution to metabolic diseases and a broad spectrum of biomedically relevant quantitative traits.

## Results

### High-depth WGS dataset of the ChinaMAP

The ChinaMAP is based on three large-scale cohorts: The China Noncommunicable Disease Surveillance 2010, a nationally representative study with 150,000 participants;^14^ the Risk Evaluation of cAncers in Chinese diabeTic Individuals: a lONgitudinal (REACTION) study with 250,000 participants^15^ and the Community-based Cardiovascular Risk During Urbanization in Shanghai with 50,000 participants.^16^ A wide variety of phenotypic information, as well as biological samples, has been collected for each of ~450,000 participants. These cohorts are followed periodically for new cases of diseases and disease complications. In the first phase of the ChinaMAP, we have randomly selected participants from 8 ethnic populations (Han, Hui, Manchu, Miao, Mongolian, Yi, Tibetan and Zhuang) across 27 provinces of China without biased selection or filtration, and completed the analysis of deep WGS data (40.80×) from 10,588 participants. The mean baseline age was 54 years and 64.8% were women (Supplementary information, Fig. S1a and Table S1).

**Fig. 1:**
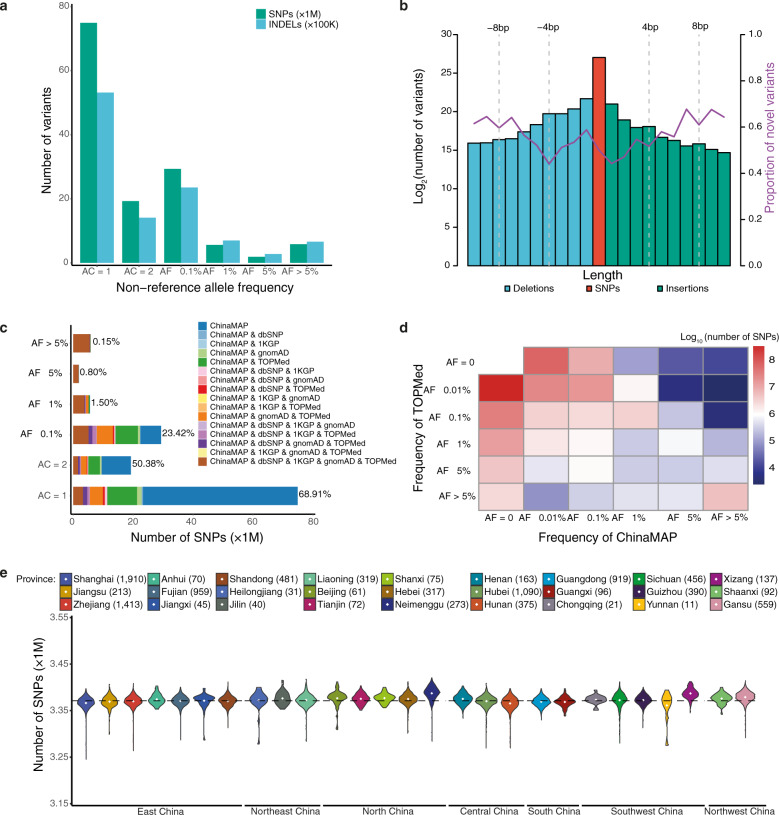
Distribution and patterns of genetic variants from the ChinaMAP. **a** The number and allele frequency spectrum of autosomal variants (SNPs and INDELs). **b** The length and number description of autosomal variants. The purple line indicates the proportion of novel variants. **c** The number and allele frequency spectrum of known and novel SNPs identified in the ChinaMAP compared with the TOPMed, gnomAD, dbSNP, and 1KGP databases. **d** Comparison of the frequency distribution of SNPs between the TOPMed and ChinaMAP. **e** The number of autosomal SNPs identified cross the 7 large geographical areas and 27 provinces.

High-depth WGS data (> 30×) is necessary for accurate detection of extremely rare variants.^17^ The ChinaMAP obtained a more massive Chinese genomic dataset compared to the low-depth genome data from non-invasive prenatal testing and SG10K study.^11,12^ The final database contained 136.75 M single nucleotide polymorphisms (SNPs) and 10.70 M insertion-deletion polymorphisms (INDELs) after stringent quality control filtering (Fig. 1a, b; Supplementary information, Table S2). The C:G > T:A (38.82%) transitions are the majority in the mutation spectrum, followed by the T:A > C:G (27.32%) transitions (Supplementary information, Fig. S1b). 2.61% of high-quality SNPs are multiallelic. Consistent with previous databases,^5,6,8,10^ the ChinaMAP data revealed that the rare variants (allele frequency (AF) < 1%) are dominant (94.16%) and 54.41% of the total are singletons (variants in only one individual). The ChinaMAP dataset has a total of 1.78 M protein-coding variants (1.21%), including 980,726 nonsynonymous, 532,701 synonymous, 27,967 stop gain/stop lost, 2851 start lost, 187,758 splice, 31,585 frameshift and 15,733 in-frame variants (Supplementary information, Table S3). The remaining 98.79% of variants are noncoding variants, for which there is still a lack of functional analysis and annotation. The quantity distribution and density of autosomal SNPs were analyzed (Supplementary information, Fig. S1c).

To ensure the information from the ChinaMAP are available to researchers, we established the ChinaMAP browser (www.mBiobank.com) for investigations as other large-scale human genomic sequencing projects, such as the DiscovEHR browser (http://www.discovehrshare.com)^6^ and the Bravo browser (https://bravo.sph.umich.edu).^10^ The summary information from the databases, including the position, reference allele, mutated allele and allele frequencies of all variants could be accessed through the ChinaMAP browser. All variants could be inquired by gene symbol, rs ID, genomic region or position. The exact number of alleles, allele frequency data in different ethnic groups and data quality for each variant from the ChinaMAP could be searched on the mBiobank website.

To analyze the novel genetic characteristics and information of the Chinese population, we compared the ChinaMAP dataset to the TOPMed (freeze 5, 463 M variants), gnomAD (v2.0.2, 125,748 exomes and 15,708 genomes), dbSNP (v149) and 1KGP. The ChinaMAP dataset exhibited great differences compared to the combination of TOPMed, gnomAD, dbSNP and 1KGP (Fig. 1c, d; Supplementary information, Fig. S2a–d). Although the sequence of East Asian population had been included in these reference databases, a large number of novel common variants (9,033 SNPs and 16,470 INDELs, AF > 5%) and low-frequency variants (15,615 SNPs and 14,581 INDELs, AF = 1%–5%) were identified in the ChinaMAP (Supplementary information, Table S4). A total of 68.3 M SNPs and 5.6 M INDELs are novel variants, the majority of which are singletons (75.3%). Furthermore, the distribution of individual variant numbers showed significant geographical and ethnic characteristics (Fig. 1e; Supplementary information, Fig. S2e, f). China has seven large geographical areas, including North, Northeast, East, Central, South, Southwest and Northwest China. The 8 ethnic groups in the ChinaMAP (Han, Zhuang, Hui, Manchu, Miao, Yi, Tibetan and Mongolian) are top-ranked by the population of Chinese ethnics. Our data showed that the Han populations from the Hexi-Corridor Area in Northwest China (Gansu province), which is a key region for the Silk Road and migration of ancient ethnic groups in history,^18^ have noticeably more individual variants (Fig. 1e). Ethnic minorities, Tibetan, Mongolian and Hui populations, have a higher level of mean individual variants than the average, whereas the Miao individuals showed an overall decreased level of variants (Supplementary information, Fig. S2f). For each individual, the median variants contained 3.37 M SNPs and 0.35 M INDELs, and the transition/transversion ratio (Ti/Tv) is 2.11 (Supplementary information, Fig. S3a, e, and Table S3). The heterozygous/homozygous ratio in Hui and Mongolian people is higher than the average (Supplementary information, Fig. S3b, e). The number of individual singletons is characterized by the geographic divisions (Supplementary information, Fig. S3c, d) and ethnic groups (Supplementary information, Fig. S3e) and distinctly divided by related and unrelated individuals (Supplementary information, Fig. S3f). The singleton variants in Miao people are less than the average (Supplementary information, Fig. S3e). Taken together, these genomic analyses revealed the genetic characteristics, diversity and complexity of the multi-ethnic Chinese population in large geographical areas.

**Fig. 2:**
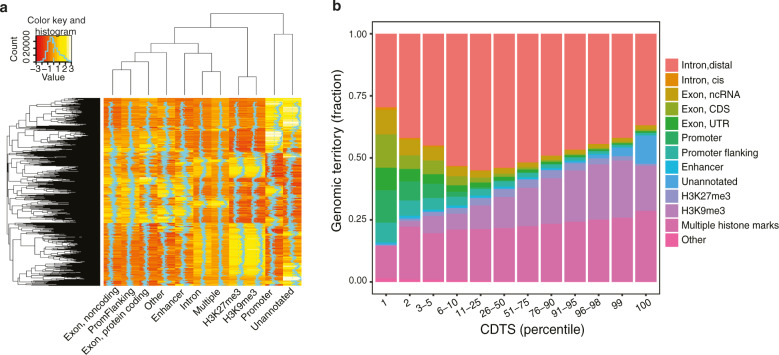
CDTS for genomic elements. **a** The relative sequence composition (*k*-mers) for different genomic elements is shown by red and yellow shaded heatmap. Heptamers are presented in rows and ordered by hierarchical clustering. The relative abundance of each heptamer is compared horizontally across the genomic elements, with the shades of red and yellow reflecting the *z* scores. **b** The cumulative territory fraction covered by each element family is shown by the bar plot in different percentile slices (1–100) based on the rank of CDTS values. The ensemble element families that were not included in substantial parts of the genome are represented as others.

To analyze the conservation of noncoding genome sequence and variants, we calculated the difference of observed variation from expected variation by the context-dependent tolerance score (CDTS) and ranked every 550 bp sliding window regions to study the context-dependent constrained regulatory regions using 16,384 unique heptamers (7-nt motifs) in the human genome.^19^ Our results showed the strong functional enrichment for non-coding variants in regulatory regions such as promoter and enhancer, similarly as reported (Fig. 2a, b).

### Loss-of-function and pathogenic variants

**Fig. 3:**
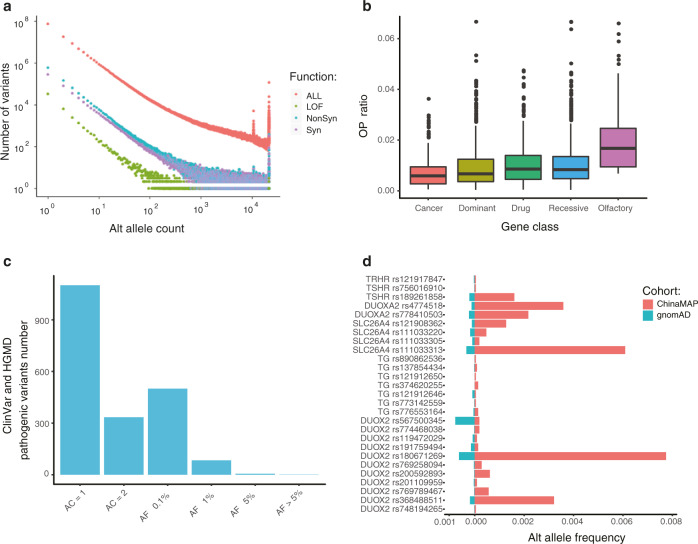
Distribution of functional and pathogenic variants. **a** Relationship between the alternate allele count and the number of variants in categories (LOF, nonsynonymous, and synonymous). **b** The OP ratio of LOF variants in functional categories of genes (cancer, genes in the Cancer Gene Census catalogs; drug, genes in the PharmGKB database; olfactory, genes related to olfactory; dominant and recessive, autosomal dominant and recessive disease-associated genes in the OMIM database). **c** The allele frequency spectrum of disease-causing variants in the HGMD and pathogenic or probably pathogenic variants in the ClinVar. **d** The allele frequencies of pathogenic variants in genes associated with hypothyroidism (*DUOX2*, *DUOXA2*, *SLC26A4*, *TG*, *TRHR* and *TSHR*) in the ChinaMAP and gnomAD.

The identification and frequency spectrum of deleterious pathogenic and predicted loss-of-function (pLOF) variants contribute to the crucial reference for Mendelian disorders. The ChinaMAP dataset contains 82,969 pLOF variants, including 48,163 SNPs and 34,806 INDELs (Supplementary information, Table S2). More than half of the pLOF variants are novel rare variants (7631) and singletons (38,490). The total of 792 common and 424 low-frequency (AF > 1%) pLOF variants included 21 novel variants. The majority of protein-coding genes (15,048 in 18,502 known genes, 81.3%) have rare pLOF variants (AF < 1%) in at least one participant (Supplementary information, Table S5). In addition, the analysis of ‘human gene knockouts’^20^ revealed that 627 genes and 29 LOF intolerant genes contained homozygous rare pLOF variants in at least one participant, which could contribute to human population-based data of gene functions (Supplementary information, Fig. S4a and Tables S5 and 6). The count numbers and spectrum of allele frequencies showed that the pLOF variants were much fewer than others under the negative selection (Fig. 3a and Supplementary information, Fig. S4b).^9,21^ The OP (observed to potential) ratio of predicted truncating mutations indicated that the natural selection restrained pLOF variants were less tolerant with the increase of allele frequency (Supplementary information, Fig. S4c). The pLOF variants in cancer and autosomal dominant disease-associated genes were more intolerant than the variants in olfactory receptor genes, drug target genes and autosomal recessive disease-associated genes (Fig. 3b). The fractions of LOF, synonymous and nonsynonymous variants under the selection in the 1KGP, EAS (East Asian), CHB (Chinese Han in Beijing) & CHD (Chinese in Denver, United States) and ChinaMAP database were similar (Supplementary information, Fig. S4d), and the ratio of pLOF to synonymous variants in the ChinaMAP was higher (Supplementary information, Fig. S4e).

To assess the characteristics and distribution of causal variants for Mendelian disorders in the ChinaMAP, we filtered the pathogenic variants with the annotation of ClinVar database (20180603)^22,23^ and HGMD (Human Gene Mutation Database, 2016.2),^24^ and further analyzed the disease-causing variants following the guidelines from the ACMG (American College of Medical Genetics and Genomics).^25^ A total of 2026 variants or 1619 variants in the HGMD DM set were annotated as pathogenic or likely pathogenic by the ClinVar or ACMG, respectively (Fig. 3c and Supplementary information, Table S7). The candidate pathogenic variants should be defined and interpreted by further clinical and functional investigations. The pathogenic variant with the highest allele frequency in the ChinaMAP was identified as *SERPINB7* rs142859678 (AF = 0.011). The rs142859678 in *SERPINB7* (AF = 5.16 × 10^−4^, gnomAD) causes the autosomal recessive disease Nagashima-type palmoplantar keratosis, which is reported in Chinese and Japanese populations.^26^ We also identified that *SPINK1* rs148954387 (AF = 5.38 × 10^−3^), a variant that leads to chronic pancreatitis,^27^ had a higher frequency in China (AF = 2.99 × 10^−4^, gnomAD). Moreover, we noticed that the pathogenic variants in 6 genes (*DUOX2*, *DUOXA2*, *SLC26A4*, *TG*, *TRHR*, and *TSHR*) related to thyroid function were more common in the ChinaMAP than gnomAD (Fig. 3d and Supplementary information, Table S8). We found 12 individuals with homozygous or two heterozygous mutations in these genes (Supplementary information, Table S9), which could cause congenital hypothyroidism.^28^ These genetic epidemiology findings revealed the potential importance of genetic testing screening for Mendelian disorders with high incidence. The frequency spectrum of variants in the ChinaMAP (Supplementary information, Table S^7^) provides an additional reference for the studies of variants of uncertain significance (VUSs).

### Genetic diversity and the population structure

**Fig. 4:**
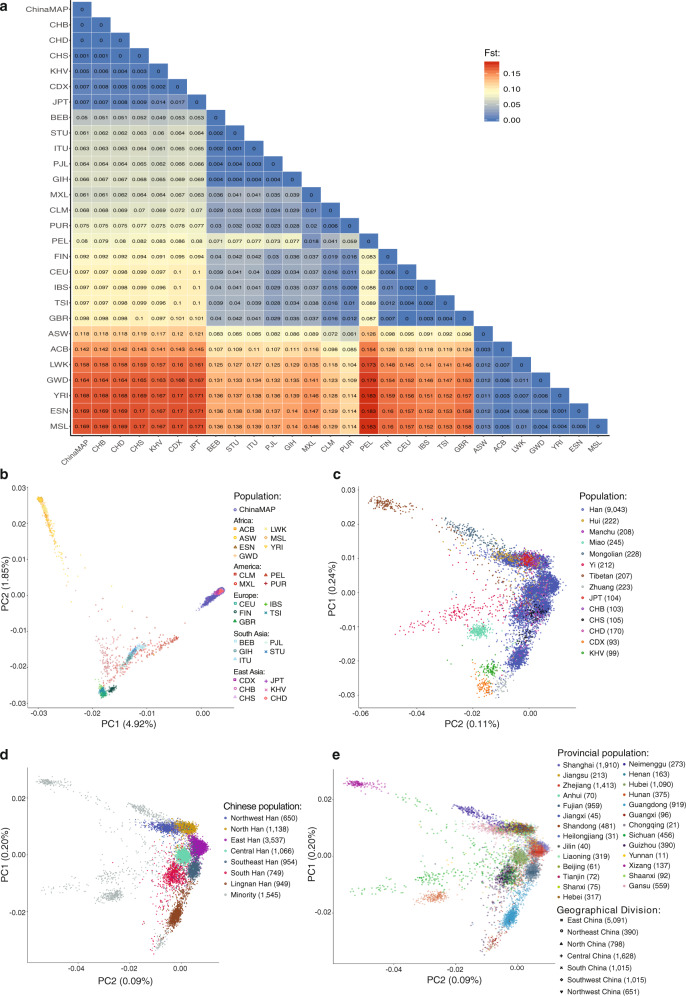
Population structure and genetic diversity analysis. **a** Pairwise Fst differences between the Chinese and other populations of European, African, South Asian, Admixed American and Latino ancestries. **b** PCA of the ChinaMAP and other populations in the 1KGP and HapMap project. **c** PCA of East Asians in different geographical areas. **d** PCA of the Chinese Han population. **e** PCA of the Chinese populations in 27 provinces and 7 geographical divisions.

The precise analysis of the population structure of the world’s largest ethnic group Chinese Han and minority ethnic groups is critical for the discovery of population genetic diversity and characteristics in East Asia. The comparative analysis of the Chinese and the world’s other populations might provide novel insights into ancestral origins and relationships of ethnic groups. Therefore, we performed principal component analysis (PCA) of the 10,588 participants in the ChinaMAP with the 1KGP and HapMap project^6,29^ as a reference to distinguish the ethnic and geographic ancestry of Chinese and other populations. The PCA and pairwise Fst calculation showed great differences between the Chinese population and European, African, South Asian, Admixed American and Latino ancestries (Fig. 4a, b). The African ancestry and Chinese population showed the largest genetic distance (MSL, Sierra Leone, Fst = 0.15; ESN, Nigeria, Fst = 0.15; YRI, Nigeria, Fst = 0.149). The genetic structures of Chinese, Japanese and Kinh Vietnamese populations of East Asian ancestry, are very similar (JPT, Japan, Fst = 0.007; KHV, Vietnam, Fst = 0.005).

**Fig. 5:**
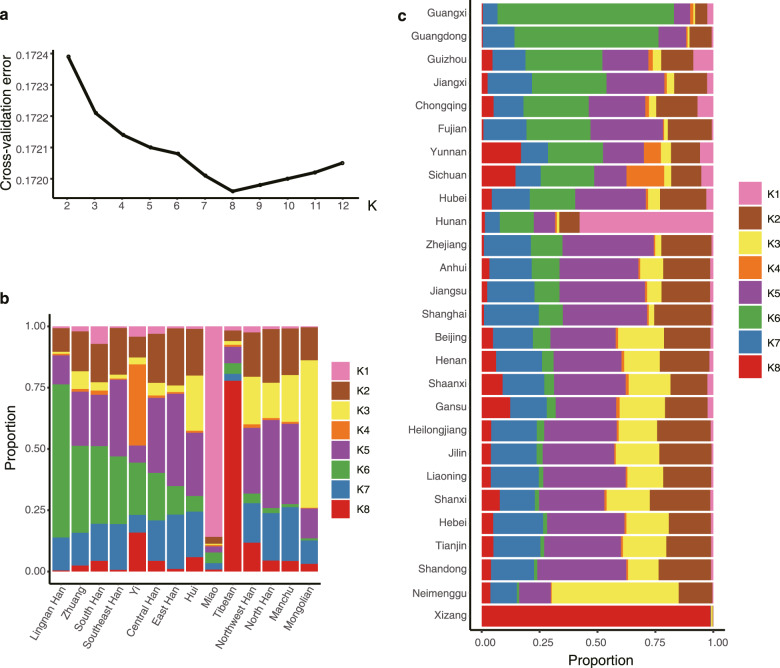
Population structure analysis by the admixture program. **a** Estimation of the number of groups (ranging from 2 to 12) for K values in ADMIXTURE. **b** Distribution of the 8 ancestry components in 7 Chinese Han populations and 7 ethnic minorities as inferred using the admixture program for K = 8. **c** Geographic distribution of the 8 ancestry components using the admixture program for K = 8. Each bar plot represents the average ancestry proportions across individuals from the indicated province.

Furthermore, the PCA of geographical and ethnic groups in East Asian ancestry showed difference and clustering of different populations. Referring to the SNP references, the CHB and CHS (Southern Han Chinese) populations are mainly clustered with Han in North China and South China, respectively, and the majority of CHD could be migrants from Han population in the east and south coastal provinces (Zhejiang, Fujian, and Guangdong). The Japanese individuals (JPT) are overlapped with Chinese Han populations in North China (Fig. 4c). Chinese ethnic minorities, Tibetan, Yi, Mongolian, Miao, Zhuang, and CDX (Chinese Dai in Xishuangbanna) populations, and Kinh Vietnamese (KHV) in East Asian have unique clusters (Fig. 4c). The Chinese Han population could be mainly distinguished into 7 population clusters, including Northwest Han (Gansu, Shaanxi), North Han (Beijing, Tianjin, Henan, Hebei, Shandong, Liaoning, Jilin, Heilongjiang and Shanxi), East Han (Jiangsu, Zhejiang, Shanghai and Anhui), Central Han (Hubei), Southeast Han (Fujian), South Han (Guizhou, Sichuan, Chongqing, Hunan, Yunnan, Jiangxi) and Lingnan Han (Guangdong, Guangxi) (Fig. 4d, e). Manchu and a part of Zhuang populations are genetically clustered with North Han, which is consistent with the historical population migration. Hui population is clustered with Northwest Han in the Hexi-Corridor Area (Fig. 4c, d). Moreover, we investigated the Chinese population structure using ADMIXTURE with a model of 8 hypothetical ancestral components (K = 8) selected by cross-validation (Fig. 5a). The proportion and distribution of the eight ancestry components in the individuals from 7 Chinese Han populations and 7 ethnic minorities in 27 provinces, confirmed the clustering of the Chinese Han population in different regions and genetic characteristics of ethnic minorities (Fig. 5b, c; Supplementary information, Fig. S5). Altogether, our findings provided the precise genetic structure of Chinese Han and minority ethnic populations, revealing the genomic diversity and distribution of the Chinese population.

### Polygenic risk score and WGS association analyses

The advance of deep WGS data and the diversity of Chinese population empower the ChinaMAP for the discovery of novel functional rare and population-specific variants in East Asian ancestry.^30^ Therefore, we performed the polygenic risk score (PRS) profiling for individual genetic risk estimation,^31,32^ single variant association analysis and sequence kernel association test (SKAT) for rare variant association analysis.

**Fig. 6:**
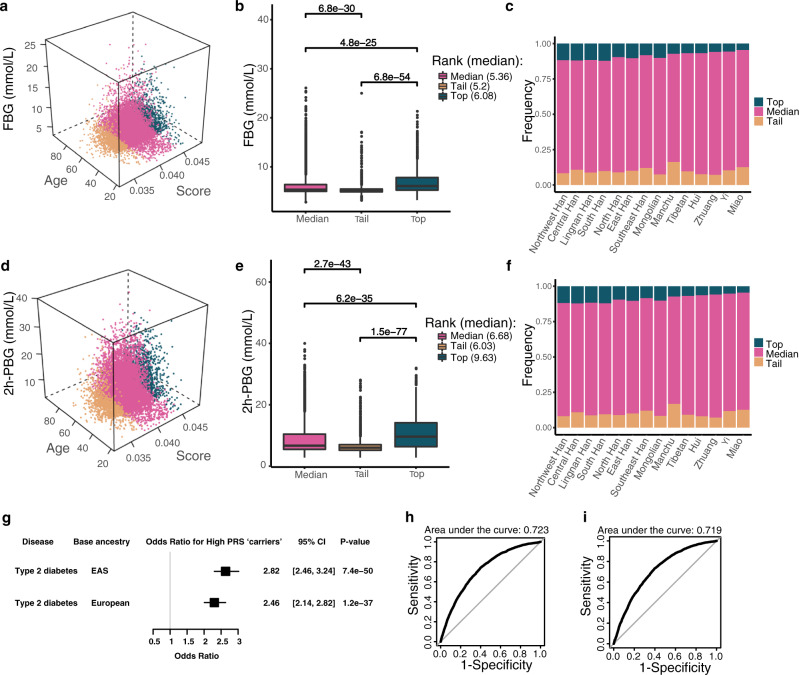
PRSs for blood glucose. **a**, **d** The three-dimensional position of age, PRS and value of FBG or 2h-PBG for each individual, which is colored by top, median and tail PRS groups. **b**, **e** Boxplot comparison of the average FBG and 2h-PBG levels from the top, median and tail PRS groups. **c**, **f** Histogram of the PRS percentage (top, median and tail) in different Chinese populations. **g** Odds ratio (OR) analysis of type 2 diabetes for the individuals with the top 10% of PRS calculated based on East Asian and European base datasets. CI, confidence interval. **h** ROC curve for risk prediction of type 2 diabetes based on East Asian populations. **i** ROC curve for risk prediction of type 2 diabetes based on European populations.

We investigated the PRSs for the most common metabolic traits, fasting blood glucose (FBG) and 2-hour postprandial blood glucose (2h-PBG). The recent large-scale meta-analysis data of GWASs from East Asian populations^33^ and European populations^34^ were used separately as base datasets for the PRS calculation of the target data from the ChinaMAP. The combination ranking of PRSs, ages and values of blood glucose showed the three-dimensional position of each individual in the whole population (Fig. 6a, d). The individuals with the top 10% of PRSs showed more severe phenotypes with aging. There were significant phenotypic differences between the top 10% and tail 10% individuals in the PRS ranking of FBG (*P* = 6.8 × 10^−54^; Fig. 6b) and 2h-PBG (*P* = 1.5 × 10^−77^; Fig. 6e). The populations from Northwest, Central, South and Lingnan Han exhibited a higher proportion of top PRS ranking compared to ethnic minorities Miao, Yi and Zhuang, indicating the diverse genetic predisposition of metabolic characteristics in Chinese Han and ethnic minorities (Fig. 6c, f). Comparison of the data from base datasets of East Asian (Fig. 6a–f) and European populations (Supplementary information, Fig. S6a–f) revealed that the PRS results based on East Asian populations were more significant and accurate. In addition, individuals with the top 10% of PRSs had significantly increased risk of type 2 diabetes (odds ratio [95% CI] = 2.82 [2.46, 3.24], *P* = 7.4 × 10^−50^). The odds ratio calculated by European base dataset was less significant (Fig. 6g). The area under the receiver-operator curve (AUC) analysis indicated that the risk prediction of type 2 diabetes was feasible (Fig. 6h, i). These findings supported the value of PRS and the importance of base datasets from East Asian cohorts for the precise individual genetic risk estimation of metabolic diseases.

**Fig. 7:**
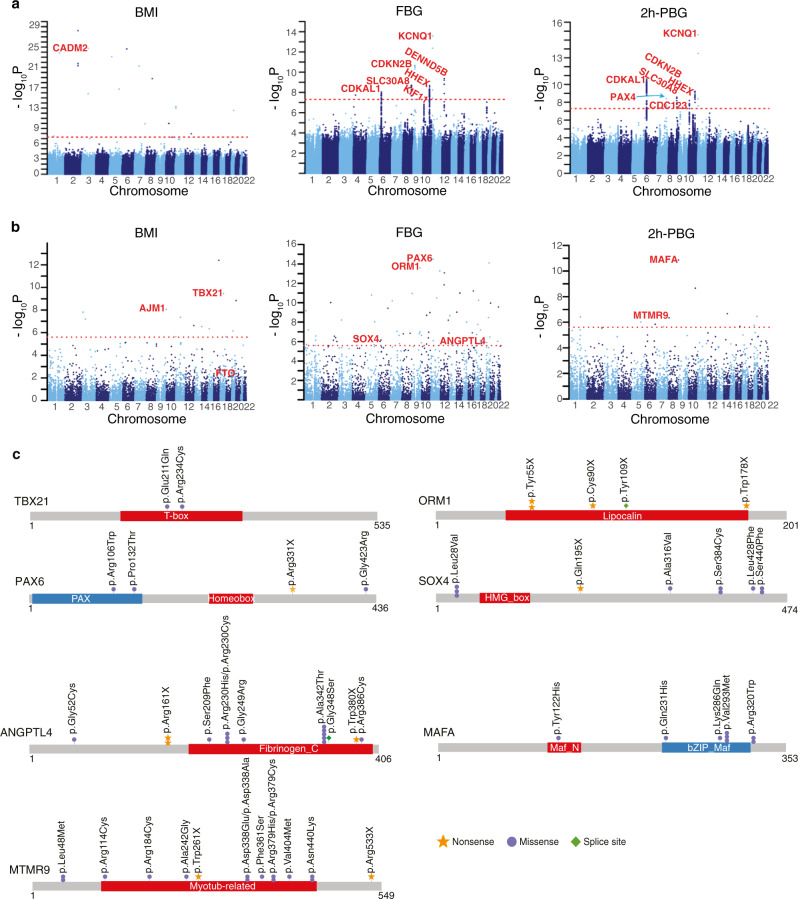
WGS association analysis of BMI and blood glucose. **a** Manhattan plots for common and low-frequency single variant association analysis of BMI, FBG and 2h-PBG. Redline is *P* = 5 × 10^−8^. **b** Manhattan plots for the gene-based association analysis of BMI, FBG and 2h-PBG. Redline is *P* = 2.5 × 10^−6^. **c** The significant signals identified in the SKAT analysis are shown in genes *TBX21*, *MAFA*, *ANGPTL4*, *PAX6*, *MTMR9*, *ORM1* and *SOX4*.

The large proportion of novel variants from the ChinaMAP data could facilitate the discovery of novel variants and genes in the WGS association analyses of metabolic traits.^35,36^ We performed single variant association analysis and SKAT analysis of BMI, FBG and 2h-PBG by the EPACTS software (Fig. 7a, b). Our results from the blood glucose analysis validated well-established gene loci associated with type 2 diabetes with common SNPs in *CDKAL1*, *SLC30A8*, *SND1-PAX4*, *IDE-KIF11-HHEX*, *CDKN2A-CDKN2B*, *KCNQ1* and *CDC123*,^33,37,38^ and identified a novel locus associated with FBG in *DENND5B* (Fig. 7a; Supplementary information, Table S10). We also identified novel Asian-specific SNPs associated with BMI (rs369036035, *P* = 1.72 × 10^−25^ and rs372115169, *P* = 1.55 × 10^−16^) in *CADM2* (Fig. 7a; Supplementary information, Table S11), which mediates synaptic signaling in the brain and regulates body weight and energy homeostasis.^39^ In the SKAT analysis, rare functional variants including pLOF variants and missense variants predicted to be deleterious by MetaSVM, SIFT and PolyPhen2 were analyzed (Fig. 7b, c; Supplementary information, Table S12). Interestingly, we identified that the gene *TBX21*, which encodes the immune cell transcription factor T-bet, was significantly associated with BMI (*P* = 3.5 × 10^−10^) (Fig. 7b). Consistently, the deficiency of T-bet in mice increased body weight and insulin sensitivity.^40^ We also detected a significant signal of *PLCB3* in the BMI analysis (*P* = 4.39 × 10^−8^). Novel association between the coding variant (rs35169799) of *PLCB3* and type 2 diabetes and body-fat distribution were reported recently.^41,42^ Furthermore, we identified the *MAFA* (*P* = 1.34 × 10^−11^), *MTMR9* (*P* = 4.45 × 10^−7^) and *PAX6* (*P* = 3.39 × 10^−15^), *ANGPTL4* (*P* = 1.26 × 10^−6^), and *SOX4* (*P* = 9.46 × 10^−7^) in the analysis of FBG and 2h-PBG (Fig. 7b, c). MafA, Pax6 and Sox4 are all critical transcription factors controlling insulin production and secretion in pancreatic β-cells.^43–45^ Missense mutations of *MAFA* gene were found in familial hypoglycemia or diabetes.^46^ The association between *ANGPTL4* variants and type 2 diabetes and the underlying mechanism, and the association between *MTMR9* and obesity were reported.^47,48^ In addition, *ORM1*, which encodes the key acute phase plasma protein orosomucoid 1, was markedly associated with FBG (*P* = 2.52 × 10^−14^). The circulating orosomucoid could decrease food intake and regulate energy homeostasis via leptin receptor signaling in obese and diabetic mouse models.^49^ Taken together, our findings provided novel variants and genes for candidate association of metabolic traits.

### Genetic evaluation of individual metabolic characteristics

**Fig. 8:**
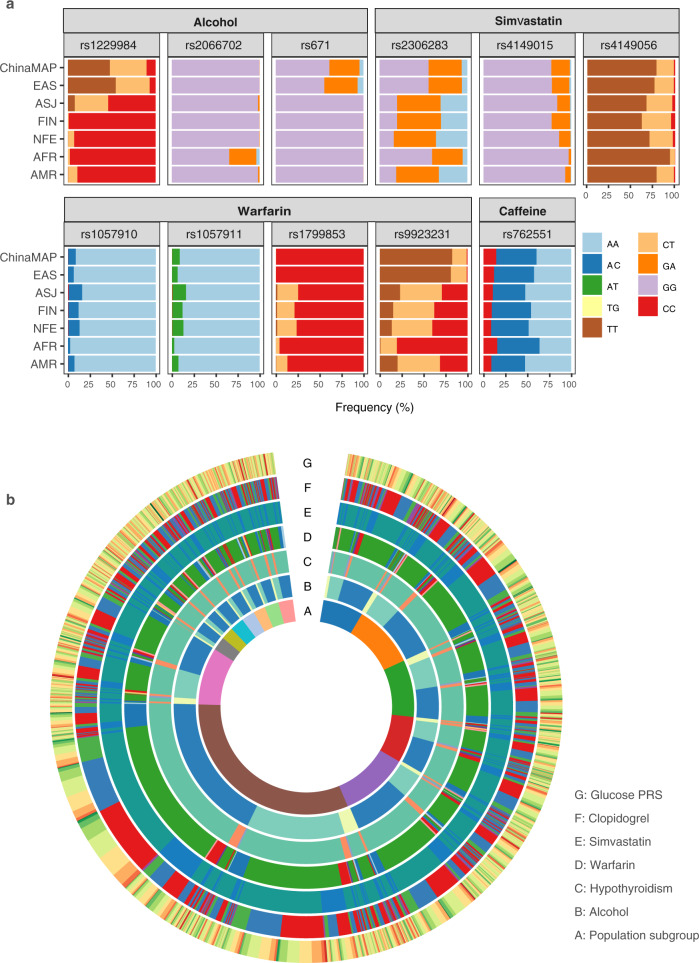
Genetic characteristics of nutrition and drug metabolism in 10,588 individuals. **a** Genotype distribution of critical SNPs for alcohol, caffeine metabolism and drug metabolism of warfarin and simvastatin. **b** Circular data for an overview of the genetic characteristics and ethnic distribution of 10,588 individuals.

Genetic evaluation and interpretation of metabolic features based on deep WGS data is a potential utility for individual health management. We explored the epidemiology and geographical characteristics of nutrition and drug metabolism in the ChinaMAP participants. Drinking alcohol and coffee are the most common dietary habits associated with health status.^50,51^ We analyzed the frequency and distribution of several critical SNPs in *ALDH2* (rs671) and *ADH1B* (rs1229984 and rs2066702) for alcohol metabolism and dependence (Fig. 8a). The data revealed that the Chinese population generally had a markedly lower clearance rate of alcohol compared to European and African ancestries (Fig. 8a; Supplementary information, Table S13). The individuals with homozygous (4.50%) and heterozygous (34.27%) *ALDH2* rs671, which is associated with the ‘Asian Blush’, have a higher risk of acetaldehyde accumulation and esophageal cancer.^50^ Geographically, the populations from the North have a stronger ability of alcohol metabolism than those from the South in China; individuals from ethnic minorities Tibetan, Mongolian and Yi are top-ranked, whereas those from Lingnan Han and Southeast Han ranked bottom (Supplementary information, Fig. S7a–c). The ability of caffeine metabolism is similar in different regions. The allele frequency of *CYP1A2* rs762551 for caffeine metabolism was comparable between the Chinese populations and other ancestries (Fig. 8a; Supplementary information, Fig. S7d).

The genetic tests for the use of anticoagulant and antiplatelet drugs are the common clinical applications of pharmacogenomics. We performed the therapeutic classification and calculated the dosage of warfarin and clopidogrel in all individuals according to the Clinical Pharmacogenetics Implementation Consortium (CPIC) Guidelines.^52,53^ The analysis of SNPs in *CYP4F2*, *VKORC1* and *CYP2C9* indicated that almost all Chinese should reduce the dosage of warfarin (Fig. 8a). The majority of individuals should have a dose reduction of ~2–3 mg/day (Supplementary information, Fig. S7e) on the basis of warfarin dosing algorithms (average 5 mg/day). The analysis of *CYP2C19* genotypes revealed that more than half of the Chinese individuals (59.08%) were intermediate (IMs, 46.02%) and poor metabolizers (PMs, 13.06%) of clopidogrel, who should consider therapies with alternative antiplatelet agents (Fig. 8a; Supplementary information, Fig. S7f and Table S14). Moreover, we examined the *SLCO1B1* variants to estimate the genetic risk of simvastatin-induced myopathy^54^ in the ChinaMAP. The results indicated that 21.20% of individuals should use a lower dose of simvastatin to control the risk of simvastatin-associated myopathies, such as rhabdomyolysis. The Tibetans and Miao individuals show a relatively lower risk (Fig. 8a; Supplementary information, Fig. S7g and Table S15). In summary, these data reminded the necessity of individual genetic testing for reducing the side-effects of common drugs in China. All of the genetic characteristics and geographical distribution of 10,588 individuals in the ChinaMAP were integrated into a circus for an overview of the genetic diversity in the Chinese population (Fig. 8b).

## Discussion

The genetic architecture of metabolic traits and variant associations for metabolic diseases are mainly from GWASs and exome sequencing studies of largely European ancestry cohorts.^1,13^ Human genomics from diverse ancestry populations are required for further understanding of the etiology of common metabolic diseases. The large-scale investigations on genetic characteristics of East Asian ancestry could promote the discovery and development of innovative risk assessment, prevention, and therapeutic strategies for metabolic diseases and complications. The population genomics of East Asian also could provide insights into the evolution and epidemiology of metabolic diseases.^3^

The ChinaMAP is based on established large cohorts across China, which represents the well-powered natural population for the investigations of factors associated with metabolic traits. The ChinaMAP has constructed a large and high-quality genomic dataset for the discovery of novel functional variants and high-impact genes and pathways in metabolic diseases. The ChinaMAP dataset exhibits great differences and contributes a large proportion of novel variants (68.3 M variants, 49.9%) compared to the combination of TOPMed, gnomAD, dbSNP and 1KGP datasets (Fig. 1), which is promising for the discovery of population-specific functional variants associated with diseases. The successful strategies in the genetic studies of specific populations had identified key genes in participants with type 2 diabetes^55^ or low plasma LDL cholesterol level.^56^ The ChinaMAP dataset could be a unique resource and reference for the investigation and identification of candidate disease-causing and disease-associated variants. Importantly, the frequency spectrum of VUSs in the ChinaMAP (Fig. 3; Supplementary information, Table S7) is also a valuable reference for the determination of causal variants of Mendelian diseases.^57,58^ The population-specific deleterious variants in the ChinaMAP might contribute to the discovery of rare high-impact variants in common metabolic diseases.

In the ChinaMAP, the population structure analysis demonstrated the complexity and features of genetic background in Chinese Han and minority ethnic groups across geographic regions (Figs. 4 and 5). The ethnic groups of East Asian ancestry in the ChinaMAP showed unique population genomic characteristics and large difference compared to other populations, as described before.^11,12^ Importantly, the ChinaMAP dataset revealed the genomic characteristics and relationships of Chinese Han and major ethnic minorities. Our data demonstrate that the Chinese Han population could be mainly distinguished into 7 population clusters, including Northwest Han, North Han, East Han, Central Han, Southeast Han, South Han and Lingnan Han (Fig. 4d). The previous classification of North and South Han (CHB and CHS) populations mainly represent a part of the Chinese Han, including North Han, Southeast Han and Lingnan Han. The PCA analysis from previous report^12^ demonstrated that the Singapore Chinese population was mainly overlapped with CHS, and our results showed that CHD was close to Chinese Han populations in east and south coastal provinces. These findings indicated the complexity and diversity of Chinese genomic characteristics, and that the current genomic dataset from Chinese populations abroad only represent the historical Chinese Han migrations from South and East China populations. Furthermore, the genetic diversity and population structures suggest that further construction of Chinese imputation reference panel would contribute to the genotype imputation quality in East Asian ancestry.

Currently, the established knowledge and guidelines related to medical genomics are mainly from Eurocentric resources, which are accepted and applied worldwide. The definition and interpretation of candidate pathogenic variants identified by databases with Eurocentric biases would require specific dataset, clinical and functional studies for East Asians. However, East Asian-specific studies are still limited due to lack of in-depth and well-phenotyped genomic database from cohort studies. The ChinaMAP provides a large and high-quality database for East Asian populations, which is beneficial for clinical investigation, validation and follow-up studies in the future. The East Asian-specific and novel variants from known disease-related genes in the ChinaMAP could be systematically investigated by future studies for genomic applications in East Asians, including clinical pharmacogenomics and genetic counseling.

The personal health management and disease risk assessment are core features for precision medicine. For the prevention and intervention of metabolic diseases, the individual-level genetic risk estimation by PRSs is a practical approach based on comprehensive genotype and phenotype database.^32^ For example, a recent study used PRS approach for precise, early risk detection of obesity based on a large cohort GWAS study.^59^ In this work, we showed that the PRS analysis was effective for individual risk evaluation of type 2 diabetes in the Chinese population. Notably, our findings showed that the PRS of Chinese population should be calculated according to East Asian-specific data by comparison of results based on GWAS studies from East Asian and European populations (Fig. 6; Supplementary information, Fig. S6). In addition, we identified and validated reported and novel gene loci associated with BMI and blood glucose through WGS association analysis (Fig. 7). The expansion of sample size and establishment of the base dataset of East Asians in the future would promote the precise clinical utility of PRS in the prevention of metabolic diseases. Furthermore, the personal and population scale genetic analysis of nutrition and drug metabolism for the ChinaMAP participants provided the individual and epidemiological information for metabolic characteristics (Fig. 8).

Collectively, the comprehensive database and genetic characterization of individuals from large well-phenotyped cohorts in the ChinaMAP could contribute to the molecular typing, prevention and individual management of metabolic diseases.

## Materials and methods

### Sample collection and DNA extraction

Genomic DNA was obtained from the metabolic biobank of the National Clinical Research Centre for Metabolic Diseases, Shanghai Clinical Center for Endocrine and Metabolic Diseases in Ruijin Hospital, Shanghai Jiao Tong University School of Medicine. DNA was prepared with QIAGEN DNeasy Blood & Tissue Kit. Informed consent was obtained from all study participants. All the protocols were approved by the Ruijin Hospital Ethics Committee, Shanghai Jiao Tong University School of Medicine.

### Library construction and WGS

Library construction and WGS were performed at BGI-Genomics. Sequence libraries for the BGISEQ-500 platform were prepared based on the BGISeq-500 library construction protocol. The qualified genomic DNA sample was randomly fragmented by Covaris technology and the DNA fragments were selected by size. The end-repair of DNA fragments was added an ‘A’ base at the 3′-end of each strand. BGISEQ-500 adapters were ligated to both ends of the A-tailed fragments, followed by amplification by ligation-mediated PCR (LM-PCR), single strand separation and cyclization. The rolling circle amplification (RCA) was performed to produce DNA Nanoballs (DNBs). The qualified DNBs were loaded into the patterned nanoarrays and processed for 100 bp pair-end sequencing on the BGISEQ-500 platform. Sequencing-derived raw image files were processed by the BGISEQ-500 base calling software with default parameters.

### DNA sequencing quality check

The SOAPnuke (v1.5.6, -n 0.05 -q 0.2 -l 12 -M 2)^60^ was used to filter dirty reads with adapter contamination, low-quality or unknown base. All the remaining reads were aligned to a human reference (GRCh38 build, from GENCODE) using BWA-MEM (Burrows–Wheeler Aligner, v0.7.16a, -k 49 -B 10 -L 10 -M -Y),^61^ and the producing result in BAM format was sorted by coordinate using Picard SortSam (v2.13.2). Finally, we used GATK (v4.beta.4)^62^ to mark duplicated reads and recalibrated the base quality scores.

All sequencing data were subjected to a series of quality control before further analysis with criteria: (1) base quality (Q30) > 80%; (2) mean sequence depth > 30×; (3) mapping rate ≥ 95%; (4) mismatch rate < 1%; (5) duplicate rate < 10%; (6) 20× coverage > 80%. In addition, mass spectrometric fingerprint genotyping of 21 common SNPs was used to verify that DNA sample and the sequencing data were from the same individual. The gender of every sample was inferred based on sequencing data by GATK TargetCoverageSexGenotyper (v4.beta.4). The inferred gender of sequencing data should be consistent with the clinical information. In total, 10,588 WGS data passed the quality control.

### Computing environment, variant calling and annotation

Three analysis platforms were used for the ChinaMAP data analysis. The same analysis pipeline was deployed on the SGE of Alibaba Cloud, the BGI HPC Cluster and BGI Online. The testing sequencing data of 50 samples were reanalyzed for 10 times on each analysis platform for consistency and stability. Discovery of germline short variants (SNPs and INDELs) was implemented according to the GATK Best Practice recommendations. We used the GATK HaplotypeCaller (v4.0.4.0) to call variants per sample and produced an intermediate file in GVCF format and consolidated GVCF files from 10,588 samples into one GVCF file using the GATK CombineGVCFs (v4.0.4.0). When we combined the GVCF files, the low-complexity regions (LCRs, covering 2% of the genome and identified by the mDust program)^63^ were ignored. Based on the combined GVCF file, the joint call was performed using the GATK GenotypeGVCFs (v4.0.4.0) with filter of the GATK Variant Filtration (v4.0.4.0). To improve the calling of INDELs, we only reserved the variants with the length ≤ 10 bp. The maximum number of alternate alleles should be ≤ 10. All high-confident variants, including splitting multiple alleles, were annotated with the SnpEff (v4.3)^64^. The dataset of variants was compared to the databases of dbSNP, 1KGP, gnomAD (WGS and WES data were combined, and the coordinates were converted to hg38 using GATK LiftoverVcf (v4.0.4.0)) and TOPMed to distinguish known and novel variants. The pathogenic classification of variants was annotated by the ClinVar^22^ (updated Jun 20180603) and HGMD^24^ (Human Gene Mutation Database, 2016.02). The probability of being LOF intolerant (pLI) for each gene was annotated by ExAC database (release 0.3). The genes with pLI ≥ 0.9 were defined as LOF intolerant genes.

### LOF variant definition and OP ratio calculation

Variants predicted to be stop codons, essential splice site-disrupting, initiator codon, start lost, transcript ablation and frameshift variants are defined as LOF variants. The OP ratio is a gene-based metric to quantify LOF variation while accounting for transcript size and is a useful tool for comparing the rate of LOF variation in different gene groups. It is designed to measure a gene’s tolerance to damaging amino-acid changes. The OP ratio was calculated by comparing the observed and the potential numbers of LOF sites based on dbNSFP database.^65,66^

### Estimation of natural selection

The site frequency spectrum (SFS) was calculated by counting the number of variants that exist in *i* for *i* = 1, 2,…, *n*–1, in a sample of size *n*. The fraction of variants under purifying selection was calculated by the python scripts^67^ using LOF, non-synonymous and synonymous SFS, respectively. Intron and intergenic sites were used as a reference. Variant frequency data of other populations were obtained from IKGP (*n* = 2504 for all races, *n* = 504 for East Asian and *n* = 208 for CHB and CHS), TOPMed and gnomAD.

### Population structure analysis

PCA was performed using a subset of autosomal bi-allelic SNPs. Several restrictions were employed to select the final 1,409,151 SNPs for PCA analysis, including minor allele frequency (MAF) ≥ 1% (common and low-frequency variants), genotyping rate ≥ 90%, Hardy-Weinberg-Equilibrium (HWE) *P* > 0.000001, and removing one SNP from each pair with *r*^2^ ≥ 0.5 (in windows of 50 SNPs with steps of 5 SNPs). The PCA was performed with the final SNPs using PLINK^68^ (v1.9) and EIGENSOFT^69,70^ (v7.2.1). When compared to 1KGP and CHD population in HapMap, the overlapping 124,900 SNPs between the ChinaMAP, 1KGP and CHD in HapMap Project were used for PCA analysis. Restricting PCA of CHD in HapMap, EAS populations in 1KGP and ChinaMAP was based on the overlapping 124,900 SNPs.

We also used the ADMIXTURE^71^ (v1.3.0) to estimate the individual ancestries, with the number of ancestral component K values ranging from 2 to 12. To obtain the optimal K value, we divided our data into 5 roughly equal parts. For each k = 1, 2,…5, we fitted the model with parameter λ to the other 4 parts, giving $\hat \beta ^{ - k}(\lambda )$, and computed its error in predicting the kth part: $E_k\left( \lambda \right) = \mathop {\sum}\nolimits_{i \in kthpart} {(y_i - x_i\hat \beta ^{ - k}(\lambda ))^2}$. The five-fold cross-validation error was computed: ${\mathrm{CV}}\left( \lambda \right) = \frac{1}{5}\mathop {\sum}\nolimits_{k = 1}^5 {E_k(\lambda )}$. Using the above formulas, we chosen the optimal K value that makes CV(λ) smallest. We calculated the mean pairwise Fst differences between different population groups in the HapMap and ChinaMAP cohorts by using EIGENSOFT (v7.2.1).

### Familial relationship of individuals

The relatedness of individuals was analyzed by the genotypes for 1,409,151 SNPs of each sample. SNPs were the same as in the ChinaMAP PCA analysis. Relatedness of the samples were measured by IBD (Identical by Descent) using PLINK^68^ (v1.9). Unrelated participants were identified using the proportion of relatives of PI_HAT < 0.1875. A total of 9847 unrelated participants without family relationships were determined in the ChinaMAP.

### PRS analysis

We performed PRS calculations on individual blood glucose using the PRSice software.^72^ Two independent GWAS datasets were used for PRS calculation: (1) results from a GWAS study for type 2 diabetes including 433,540 East Asian individuals;^33^ (2) results from a GWAS study^34^ for type 2 diabetes (898,130 individuals of European ancestry), and we only used the comparable variants in a GWAS study from Japanese population.^73^ We evaluated 5 main approaches to generate weighted PRSs: (1) converting genome coordinates from hg19 to hg38 for GWAS datasets; (2) only inclusion of genome-wide significant variants (*P* < 5 × 10^−8^); (3) removing linkage disequilibrium (LD); (4) exclusion of A/T and C/G SNPs to minimize errors from strand flips; (5) adjusting by age, age^2^, gender and the first two principal components of ancestry. We labeled the top 10% PRS of individuals as the top group, the last 10% PRS of individuals as the tail group, and the remaining intermediate PRS of individuals as the median group. We used the two-tailed *t*-test to compare the differences between the top, median and tail groups. The relationship of the top group with type 2 diabetes was determined using logistic regression. The AUC was calculated to assess the performance of the binary trait.

### Genotype-phenotype association analysis

The measurement and collection of phenotype information for all individuals are described previously.^14,16,63^ Before genotype-phenotype association analyses, all variants were subjected to a series of quality control with criteria: (1) median depth > 8; (2) within LCRs (< 7 single base repeat units); (3) homozygous variants (AF ≥ 0.90); (4) homozygous variants (AF ≥ 0.2); (5) genotyping rate ≥ 90%; (6) HWE > 0.000001. 4,764,593 SNPs passed the quality control. Genotype-phenotype association analyses were performed using the EPACTS with EMMAX (Efficient Mixed Model Association eXpedited) model (https://genome.sph.umich.edu/wiki/EPACTS). Empiric kinship matrix were based on 1,372,394 common and low linkage SNPs (retaining one SNP from each pair with *r*^*2*^ < 0.5 in windows of 50 SNPs with steps of 5 SNPs) in autosomal chromosomes. Kinship matrix was performed by EPACTS default parameters (“make-kin”). The single variant association analyses with common (MAF > 5%) and low-frequency (1% < MAF ≤ 5%) variants were performed by adjusting for age, age^2^, gender, the first two principal components of ancestry and an empirically derived kinship matrix for familial and distant relatedness. The statistical significance threshold for single variant EMMAX association analysis was 5 × 10^−8^. The SKAT analyses with rare variants (MAF < 1%) were performed using the mixed-model SKAT implementation in EPACTS. The rare variants in coding regions for analyses were selected using LOF variants and deleterious missense variants predicted by MetaSVM, SIFT and PolyPhen2. 120,262 SNPs in 17,156 genes were produced in the analysis process. The SKAT analyses were performed by adjusting for age, age^2^, gender, two principal components of ancestry and an empirically derived kinship matrix. The gonadal-specific expression genes were removed. The statistical significance threshold for each test was 2.5 × 10^−6^ (0.05/20,000).

### CDTS analysis

We used CDTS analysis, which depends on the difference between the observed and expected scores, to analyze the whole genome-wide variants. Because there are 16,384 heptamer (7-nt motifs) sequences in the genome, every nucleotide was part of a heptamer, and every single position could be used in the corresponding genome-wide computed scores. The observed regional tolerance score was the number of SNPs (AF > 0.0001) in the studied population in a defined region. In the same region, the expected regional tolerance score was the sum of the heptamer tolerance scores. All the autosomal SNPs were used for the CDTS analysis, except INDELs. Genomic regions were then ranked by their CDTSs. The lowest context-dependent tolerance to variation was the regions with the lowest rank (1st percentile). The highest context-dependent tolerance to variation was the regions with the highest rank (100th percentile). The genomic element region file was provided by the authors of CDTS method.^19^

### Pharmacogenetic analysis

Pharmacogenetic analysis was performed based on the PharmGKB database and the CPIC guidelines. For the warfarin dosing calculation, the *CYP2C9* and *VKORC1* genotypes were analyzed for the dosing algorithm of warfarin.^53^ *CYP2C9* and *VKORC1* allele definition table was downloaded from CPIC website. Warfarin pharmacogenetic dosing algorithm is the following formula: 5.6044 − 0.2614 × Age + 0.0087 × Height (cm) + 0.0128 × Weight (kg) − genotype dosing = Square root of weekly warfarin dose, in which, genotype dosing = −0.8677   × *VKORC1* rs9923231 A/G – 1.6974 × *VKORC1* rs9923231 A/A – 0.4854 × *VKORC1* genotype unknown – 0.5211 × CYP2C9*1/*2 – 0.9357 × CYP2C9*1/*3 – 1.0616 × CYP2C9*2/*2 – 1.9206 × CYP2C9*2/*3 – 2.3312 × CYP2C9*3/*3 – 0.2188 × *CYP2C9* genotype unknown.

The phenotypes of Clopidogrel metabolizer were analyzed based on the *CYP2C19* genotypes, and the algorithm for suggested clinical actions was based on *CYP2C19* genotypes with commonly tested *CYP2C19* variant alleles, *1 (“wild-type”), *2 (rs4244285, c.681 G > A), *3 (rs4986893, c.636 G > A), *4 (rs28399504, c.1 A > G), *5 (rs56337013, c.1297 C > T), *6 (rs72552267, c.395 G > A), *7 (rs72558186, c.819 + 2 T > A), *8 (rs41291556, c.358 T > C), *17 (rs12248560, c.−806C > T).^52^ *CYP2C19* phenotype and genotype table was downloaded from CPIC website. *CYP2C19* phenotypes included five metabolic types: normal metabolizer, intermediate metabolizer, ultrarapid metabolizer, rapid metabolizer and poor metabolizer.

The status of simvastatin metabolism was evaluated by *SLCO1B1* genotypes following the CPIC guideline.^54^ There are 36 *SLCO1B1* alleles of 29 SNPs. *1A and *1B are normal function alleles. *5, *15, and *17 are identified as decreased function alleles. The remaining alleles are annotated as possible, unknown or unclear function alleles.

### URLs

LCRs^74^; gnomAD^75^, v2.0.2: http://gnomad-old.broadinstitute.org/downloads; dbsnp (v149): ftp://ftp.ncbi.nih.gov/snp/; TOPMed^10^ BRAVO browser Freeze5 on GRCh38; GATK Best Practice recommendations^76^: https://software.broadinstitute.org/gatk/best-practices/workflow?id=11145; PharmGKB^77^: http://www.pharmgkb.org; Cancer Gene Census^78^: https://cancer.sanger.ac.uk/census; OMIM^64^: https://omim.org/; SnpEff^68^ (v4.3): http://snpeff.sourceforge.net; Plink^69^ (v1.9): http://www.cog-genomics.org/plink/1.9/; EIGENSOFT^70,71^ (v7.2.1): https://genome.sph.umich.edu/wiki/EPACTS; ADMIXTURE^79^ (v1.3.0): http://software.genetics.ucla.edu/admixture/index.html; EPACTS: https://genome.sph.umich.edu/wiki/EPACTS

## Supplementary information

Supplementary information, Figure S1

Supplementary information, Figure S2

Supplementary information, Figure S3

Supplementary information, Figure S4

Supplementary information, Figure S5

Supplementary information, Figure S6

Supplementary information, Figure S7

Supplementary information, Table S1

Supplementary information, Table S2

Supplementary information, Table S3

Supplementary information, Table S4

Supplementary information, Table S5

Supplementary information, Table S6

Supplementary information, Table S7

Supplementary information, Table S8

Supplementary information, Table S9

Supplementary information, Table S10

Supplementary information, Table S11

Supplementary information, Table S12

Supplementary information, Table S13

Supplementary information, Table S14

Supplementary information, Table S15

## Data Availability

The summary information from the ChinaMAP, including the position, reference allele, mutated allele and allele frequencies of all variants could be accessed through the ChinaMAP browser (www.mBiobank.com). Researchers can gain access to the data online. The sequencing data from the ChinaMAP have been deposited in the database of the National Clinical Research Centre for Metabolic Diseases in Ruijin Hospital, Shanghai, following the regulations of the Human Genetic Resources Administration of China (HGRAC). The sequencing data and information of the research participants are not publicly available to prevent the disclosure of individuals’ genetic identity. Further analysis of sequencing data will be made available for collaborating researchers upon request, dependent of the HGRAC’s approval.
