# Supplementary material for: The ChinaMAP analytics of deep whole genome sequences in 10,588 individuals
Source: Cell Res. 2020 Apr 30;30(9):717–31. doi: 10.1038/s41422-020-0322-9 (PMC7609296; doi:10.1038/s41422-020-0322-9)
Supplement: Supplementary file 1 — Supplementary information, Figure S1 [file 41422_2020_322_MOESM1_ESM.pdf]

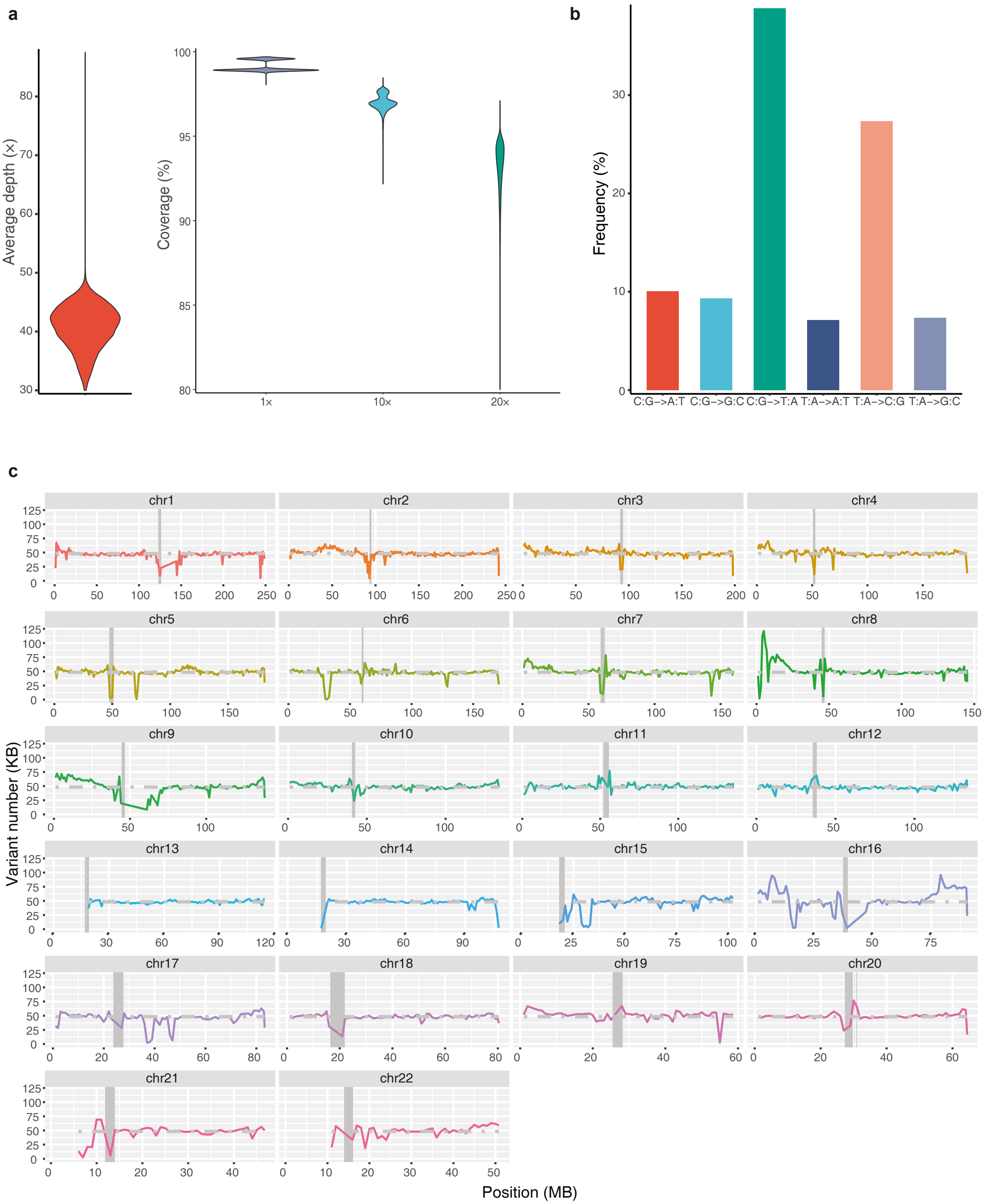
a
Average depth (×)
Coverage (%)
1×
10×
20×
b
Frequency (%)
C:G−>A:T
C:G−>G:C
C:G−>T:A
T:A−>A:T
T:A−>C:G
T:A−>G:C
c
Variant number (KB)
Position (MB)
chr1
chr2
chr3
chr4
chr5
chr6
chr7
chr8
chr9
chr10
chr11
chr12
chr13
chr14
chr15
chr16
chr17
chr18
chr19
chr20
chr21
chr22

Fig. S1 Statistical analysis of the ChinaMAP dataset. a The average sequencing depth and coverage (1×, 10×, 20×) of the ChinaMAP dataset. **b** Mutation spectrum of the autosomal SNPs. **c** The median variant number per 1 kb window (dotted grey line) in autosomal chromosomes. The grey bar region represents the centromeric region.
