# Supplementary material for: The ChinaMAP analytics of deep whole genome sequences in 10,588 individuals
Source: Cell Res. 2020 Apr 30;30(9):717–31. doi: 10.1038/s41422-020-0322-9 (PMC7609296; doi:10.1038/s41422-020-0322-9)
Supplement: Supplementary file 2 — Supplementary information, Figure S2 [file 41422_2020_322_MOESM2_ESM.pdf]

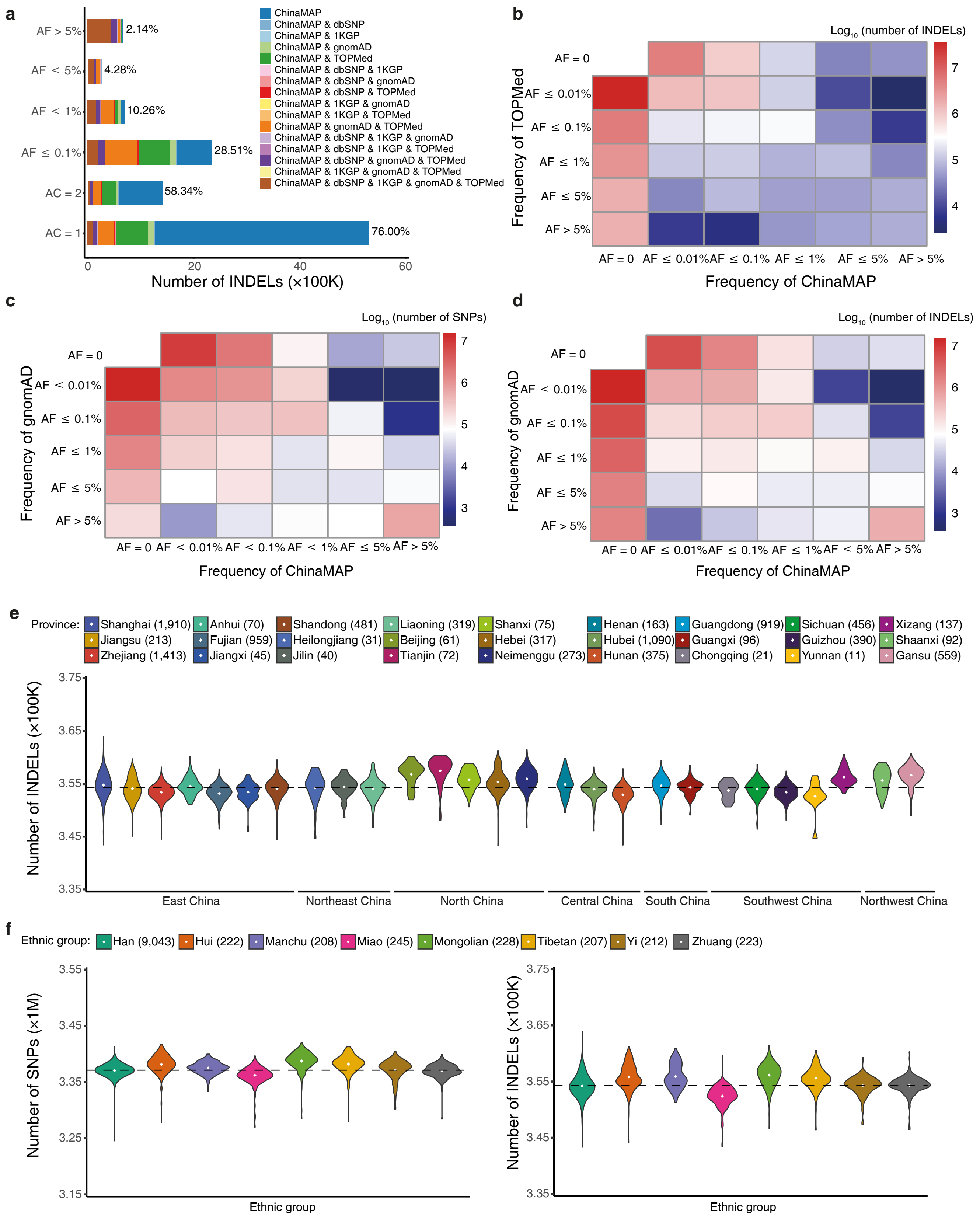

a
AF > 5%
2.14%
AF ≤ 5%
4.28%
AF ≤ 1%
10.26%
AF ≤ 0.1%
28.51%
AC = 2
58.34%
AC = 1
76.00%
0
20
40
60
Number of INDELs (×100K)
ChinaMAP
ChinaMAP & dbSNP
ChinaMAP & 1KGP
ChinaMAP & gnomAD
ChinaMAP & TOPMed
ChinaMAP & dbSNP & 1KGP
ChinaMAP & dbSNP & gnomAD
ChinaMAP & dbSNP & TOPMed
ChinaMAP & 1KGP & gnomAD
ChinaMAP & 1KGP & TOPMed
ChinaMAP & gnomAD & TOPMed
ChinaMAP & dbSNP & 1KGP & gnomAD
ChinaMAP & dbSNP & 1KGP & TOPMed
ChinaMAP & dbSNP & gnomAD & TOPMed
ChinaMAP & 1KGP & gnomAD & TOPMed
ChinaMAP & dbSNP & 1KGP & gnomAD & TOPMed
b
Log10 (number of INDELs)
Frequency of TOPMed
AF = 0
AF ≤ 0.01%
AF ≤ 0.1%
AF ≤ 1%
AF ≤ 5%
AF > 5%
7
6
5
4
Frequency of ChinaMAP
c
Log10 (number of SNPs)
Frequency of gnomAD
AF = 0
AF ≤ 0.01%
AF ≤ 0.1%
AF ≤ 1%
AF ≤ 5%
AF > 5%
7
6
5
4
3
Frequency of ChinaMAP
d
Log10 (number of INDELs)
Frequency of gnomAD
Frequency of ChinaMAP
e
Province:
Shanghai (1,910)
Anhui (70)
Shandong (481)
Liaoning (319)
Shanxi (75)
Henan (163)
Guangdong (919)
Sichuan (456)
Xizang (137)
Jiangsu (213)
Fujian (959)
Heilongjiang (31)
Beijing (61)
Hebei (317)
Hubei (1,090)
Guangxi (96)
Guizhou (390)
Shaanxi (92)
Zhejiang (1,413)
Jiangxi (45)
Jilin (40)
Tianjin (72)
Neimenggu (273)
Hunan (375)
Chongqing (21)
Yunnan (11)
Gansu (559)
Number of INDELs (×100K)
3.75
3.65
3.55
3.45
3.35
East China
Northeast China
North China
Central China
South China
Southwest China
Northwest China
f
Ethnic group:
Han (9,043)
Hui (222)
Manchu (208)
Miao (245)
Mongolian (228)
Tibetan (207)
Yi (212)
Zhuang (223)
Number of SNPs (×1M)
3.55
3.45
3.35
3.25
3.15
Ethnic group
Number of INDELs (×100K)
3.75
3.65
3.55
3.45
3.35
Ethnic group

Fig. S2 Analysis and statistics of autosomal INDELs. a The number and allele frequency spectrum of known and novel autosomal INDELs identified in the ChinaMAP compared with the TOPMed, gnomAD, dbSNP and 1KGP database. **b** The frequency distribution of INDELs between the ChinaMAP and TOPMed. **c, d** The frequency distribution of autosomal SNPs and INDELs between the ChinaMAP and gnomAD. **e** The number of autosomal INDELs identified in different provinces and geographical divisions of China. **f** The number of autosomal SNPs and INDELs identified in ethnic groups.
