# Supplementary material for: The ChinaMAP analytics of deep whole genome sequences in 10,588 individuals
Source: Cell Res. 2020 Apr 30;30(9):717–31. doi: 10.1038/s41422-020-0322-9 (PMC7609296; doi:10.1038/s41422-020-0322-9)
Supplement: Supplementary file 3 — Supplementary information, Figure S3 [file 41422_2020_322_MOESM3_ESM.pdf]

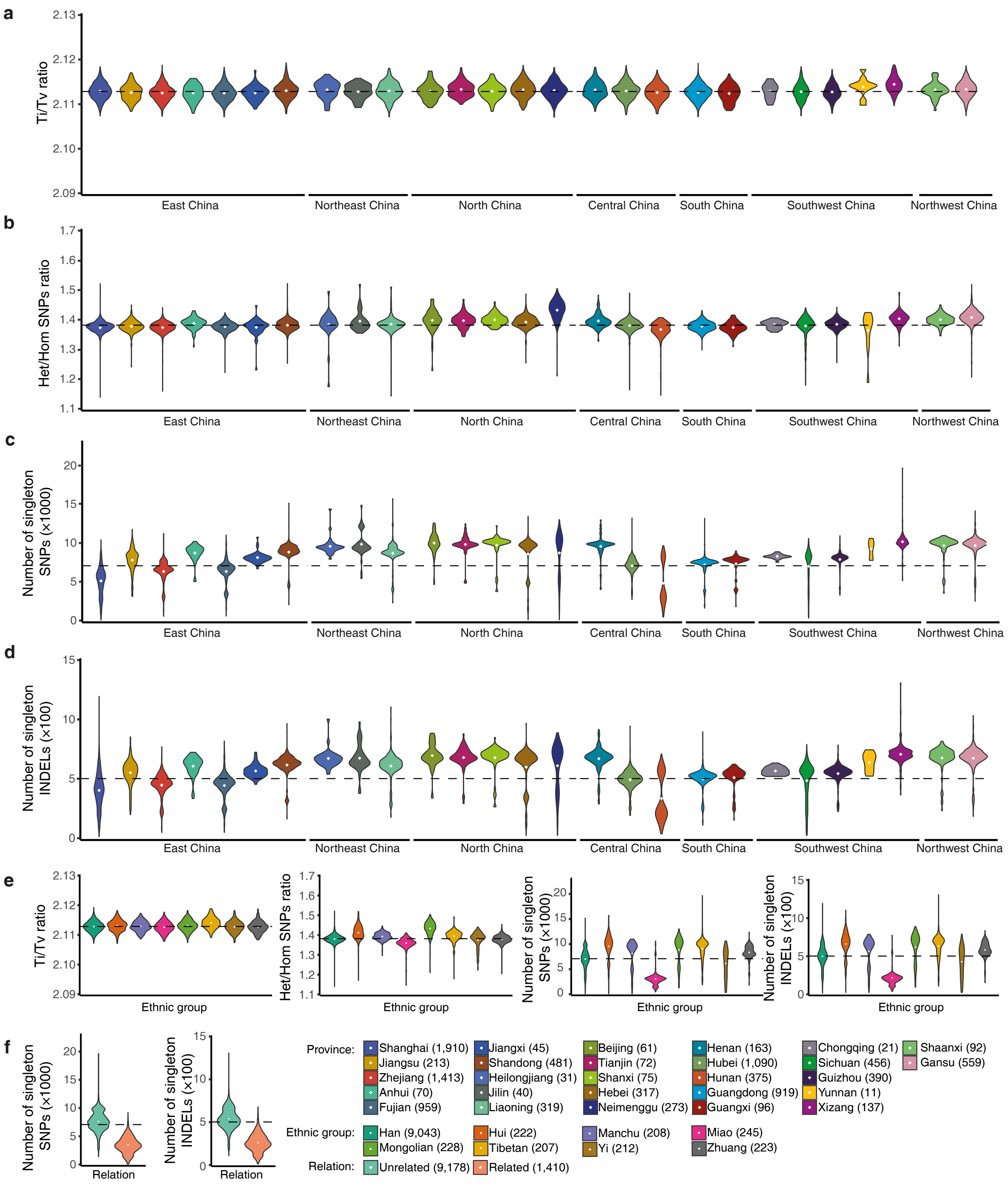

a
Ti/Tv ratio
East China
Northeast China
North China
Central China
South China
Southwest China
Northwest China
b
Het/Hom SNPs ratio
c
Number of singleton SNPs (×1000)
d
Number of singleton INDELs (×100)
e
Ethnic group
f
Relation
Province:
Shanghai (1,910)
Jiangsu (213)
Zhejiang (1,413)
Anhui (70)
Fujian (959)
Jiangxi (45)
Shandong (481)
Heilongjiang (31)
Jilin (40)
Liaoning (319)
Beijing (61)
Tianjin (72)
Shanxi (75)
Hebei (317)
Neimenggu (273)
Henan (163)
Hubei (1,090)
Hunan (375)
Guangdong (919)
Guangxi (96)
Chongqing (21)
Sichuan (456)
Guizhou (390)
Yunnan (11)
Xizang (137)
Shaanxi (92)
Gansu (559)
Ethnic group:
Han (9,043)
Mongolian (228)
Hui (222)
Tibetan (207)
Manchu (208)
Yi (212)
Miao (245)
Zhuang (223)
Relation:
Unrelated (9,178)
Related (1,410)

Fig. S3 Analysis of autosomal SNPs and INDELs in the ChinaMAP. a The ratios of transition to transversion (Ti/Tv) of autosomal SNPs identified in different provinces and geographical divisions of China. **b** The ratios of heterozygous to homozygous (Het/Hom) of autosomal SNPs identified in different provinces and geographical divisions of China. **c** The number of autosomal singleton SNPs identified in different provinces and geographical divisions of China. **d** The number of autosomal singleton INDELs identified in different provinces and geographical divisions of China. **e** The ratios of transition to transversion (Ti/Tv) of autosomal SNPs, ratio of heterozygous to homozygous (Het/Hom) of autosomal SNPs, number of autosomal singleton SNPs and INDELs identified in ethnic groups. **f** The number of autosomal singleton SNPs and INDELs identified in related and unrelated participants.
