# Supplementary material for: The ChinaMAP analytics of deep whole genome sequences in 10,588 individuals
Source: Cell Res. 2020 Apr 30;30(9):717–31. doi: 10.1038/s41422-020-0322-9 (PMC7609296; doi:10.1038/s41422-020-0322-9)
Supplement: Supplementary file 4 — Supplementary information, Figure S4 [file 41422_2020_322_MOESM4_ESM.pdf]

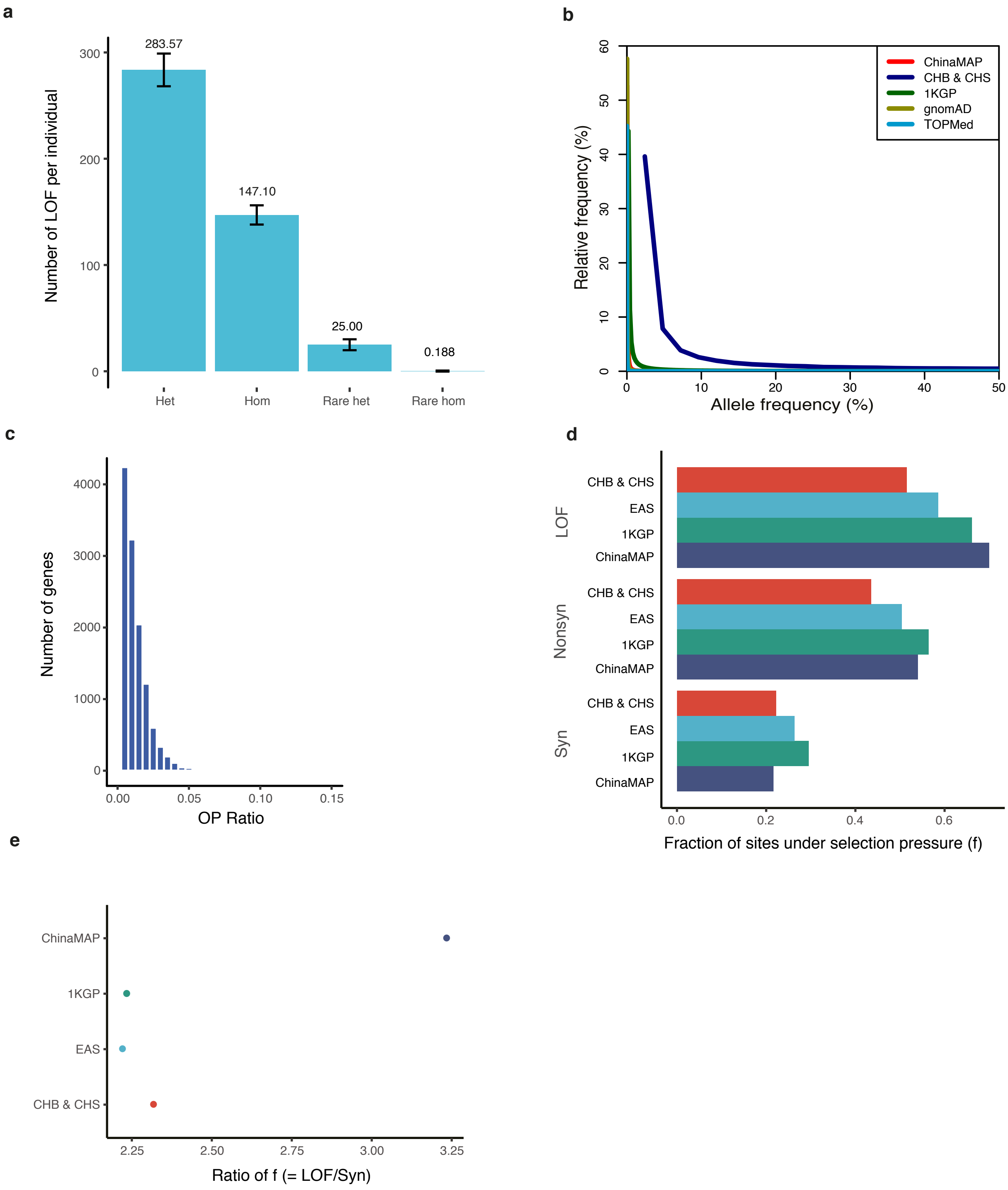

a
283.57
147.10
25.00
0.188
Number of LOF per individual
300
200
100
0
Het
Hom
Rare het
Rare hom
b
ChinaMAP
CHB & CHS
1KGP
gnomAD
TOPMed
Relative frequency (%)
60
50
40
30
20
10
0
0
10
20
30
40
50
Allele frequency (%)
c
Number of genes
4000
3000
2000
1000
0
0.00
0.05
0.10
0.15
OP Ratio
d
LOF
Nonsyn
Syn
CHB & CHS
EAS
1KGP
ChinaMAP
0.0
0.2
0.4
0.6
Fraction of sites under selection pressure (f)
e
ChinaMAP
1KGP
EAS
CHB & CHS
2.25
2.50
2.75
3.00
3.25
Ratio of f (= LOF/Syn)

Fig. S4 The functional categories and genomic regions of SNPs. a The number of loss-of-function (LOF) variants per individual. Error bars represent standard deviation. Every individual had 284 heterozygous and 147 homozygous LOF variants, of which 25 and 0.188 are rare (AF < 1%) on average. **b** Frequency spectrum for the variants of intergenic regions in the ChinaMAP, CHB & CHS, 1KGP, gnomAD and TOPMed database. **c** The distribution of observed/predicted (OP) ratio. **d** The fraction of sites under selection pressure (f) calculated for LOF, nonsynonymous and synonymous SNPs, separately. **e** The ratio of f between LOF variants and synonymous SNPs in datasets.
