# Supplementary material for: The ChinaMAP analytics of deep whole genome sequences in 10,588 individuals
Source: Cell Res. 2020 Apr 30;30(9):717–31. doi: 10.1038/s41422-020-0322-9 (PMC7609296; doi:10.1038/s41422-020-0322-9)
Supplement: Supplementary file 5 — Supplementary information, Figure S5 [file 41422_2020_322_MOESM5_ESM.pdf]

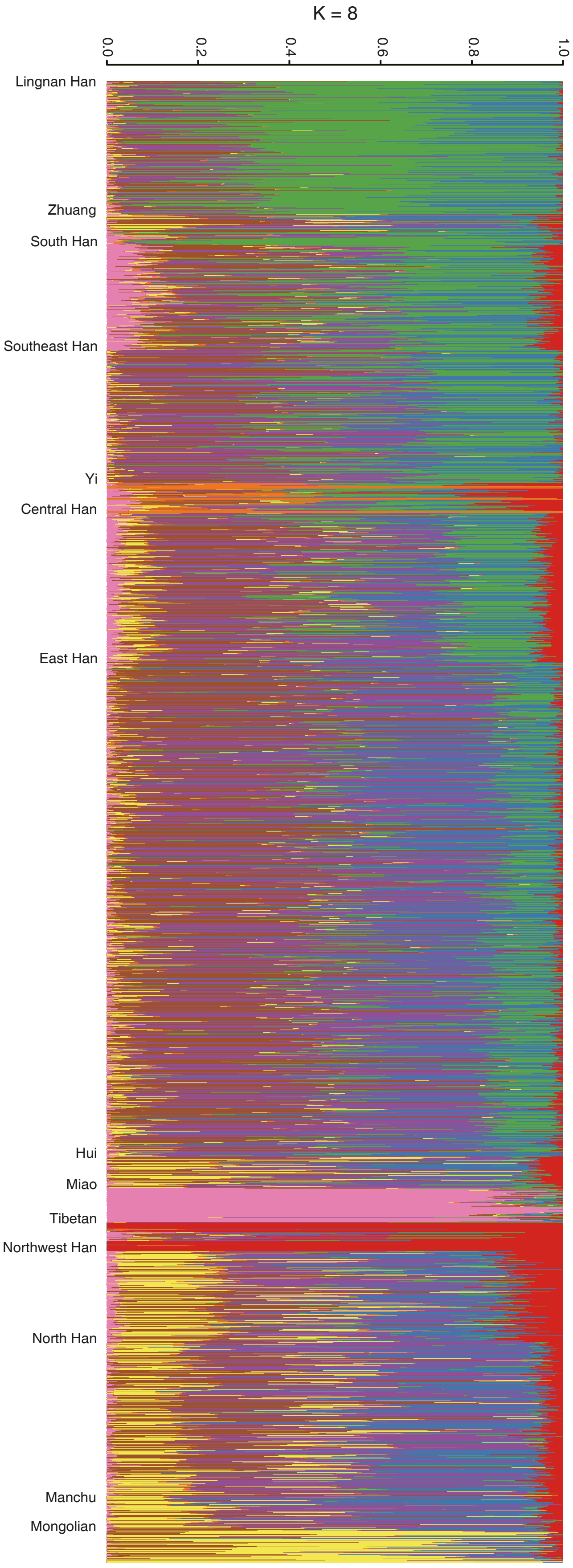

K = 8
0.0
0.2
0.4
0.6
0.8
1.0
Lingnan Han
Zhuang
South Han
Southeast Han
Yi
Central Han
East Han
Hui
Miao
Tibetan
Northwest Han
North Han
Manchu
Mongolian

Fig. S5 The population structure analysis of 10,588 individuals from 7 Chinese Han and 7 ethnic minority populations using the admixture program for K = 8.
