# Supplementary material for: The ChinaMAP analytics of deep whole genome sequences in 10,588 individuals
Source: Cell Res. 2020 Apr 30;30(9):717–31. doi: 10.1038/s41422-020-0322-9 (PMC7609296; doi:10.1038/s41422-020-0322-9)
Supplement: Supplementary file 6 — Supplementary information, Figure S6 [file 41422_2020_322_MOESM6_ESM.pdf]

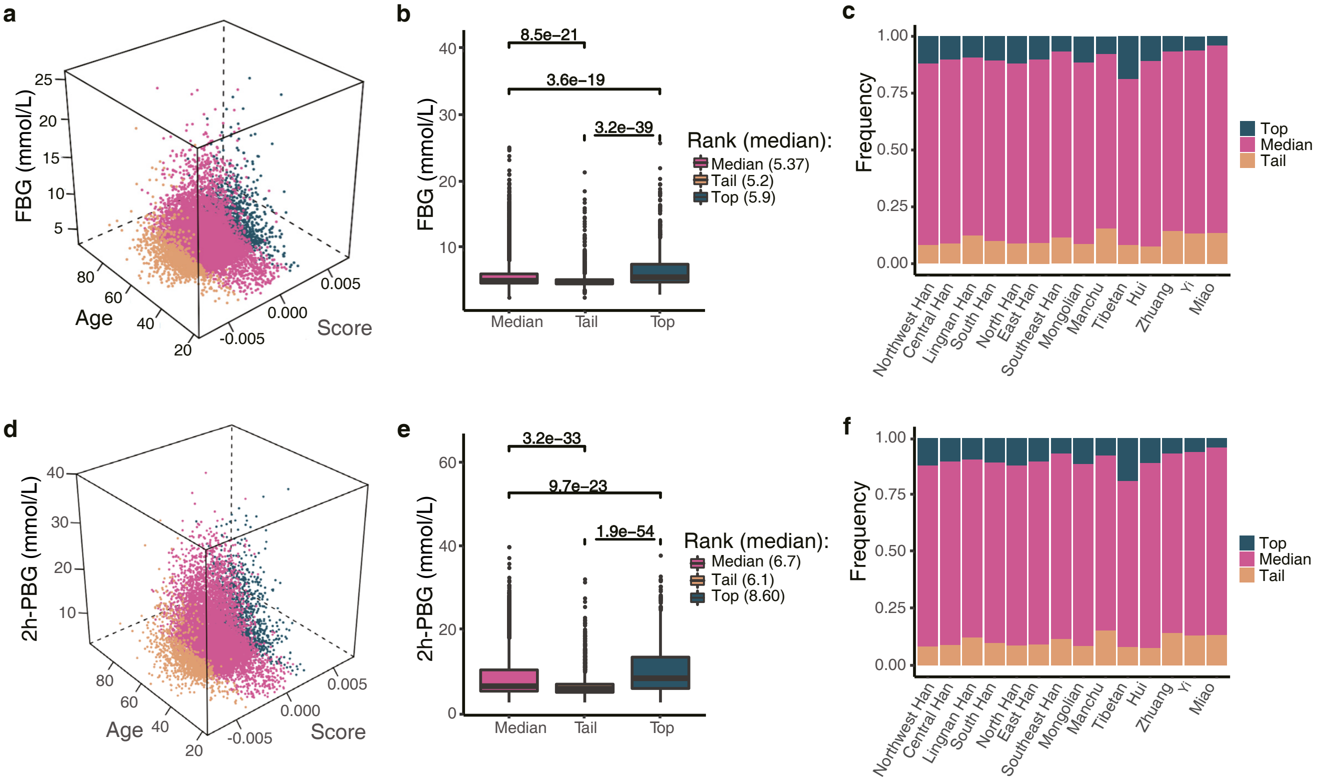
a
FBG (mmol/L)
25
20
15
10
5
80
60
Age
40
20
0.005
0.000
-0.005
Score
b
8.5e−21
3.6e−19
3.2e−39
40
30
20
10
FBG (mmol/L)
Median
Tail
Top
Rank (median):
Median (5.37)
Tail (5.2)
Top (5.9)
c
1.00
0.75
0.50
0.25
0.00
Frequency
Top
Median
Tail
Northwest Han
Central Han
Lingnan Han
South Han
North Han
East Han
Southeast Han
Mongolian
Manchu
Tibetan
Hui
Zhuang
Yi
Miao
d
2h-PBG (mmol/L)
40
30
20
10
80
60
Age
40
20
0.005
0.000
-0.005
Score
e
3.2e−33
9.7e−23
1.9e−54
60
40
20
0
2h-PBG (mmol/L)
Median
Tail
Top
Rank (median):
Median (6.7)
Tail (6.1)
Top (8.60)
f
1.00
0.75
0.50
0.25
0.00
Frequency
Top
Median
Tail
Northwest Han
Central Han
Lingnan Han
South Han
North Han
East Han
Southeast Han
Mongolian
Manchu
Tibetan
Hui
Zhuang
Yi
Miao

Fig. S6 Polygenic risk scores for blood glucose from European base dataset. a, d The three-dimensional position of age, PRS and values of FBG and 2h-PBG for each individual, which is colored by top, median and tail PRS groups. **b, e** Boxplot comparison of the average FBG and 2h-PBG levels from the top, median and tail PRS groups. **c, f** Histogram of the PRS percentage (top, median and tail) in different Chinese populations.
