# Supplementary material for: The ChinaMAP analytics of deep whole genome sequences in 10,588 individuals
Source: Cell Res. 2020 Apr 30;30(9):717–31. doi: 10.1038/s41422-020-0322-9 (PMC7609296; doi:10.1038/s41422-020-0322-9)
Supplement: Supplementary file 7 — Supplementary information, Figure S7 [file 41422_2020_322_MOESM7_ESM.pdf]

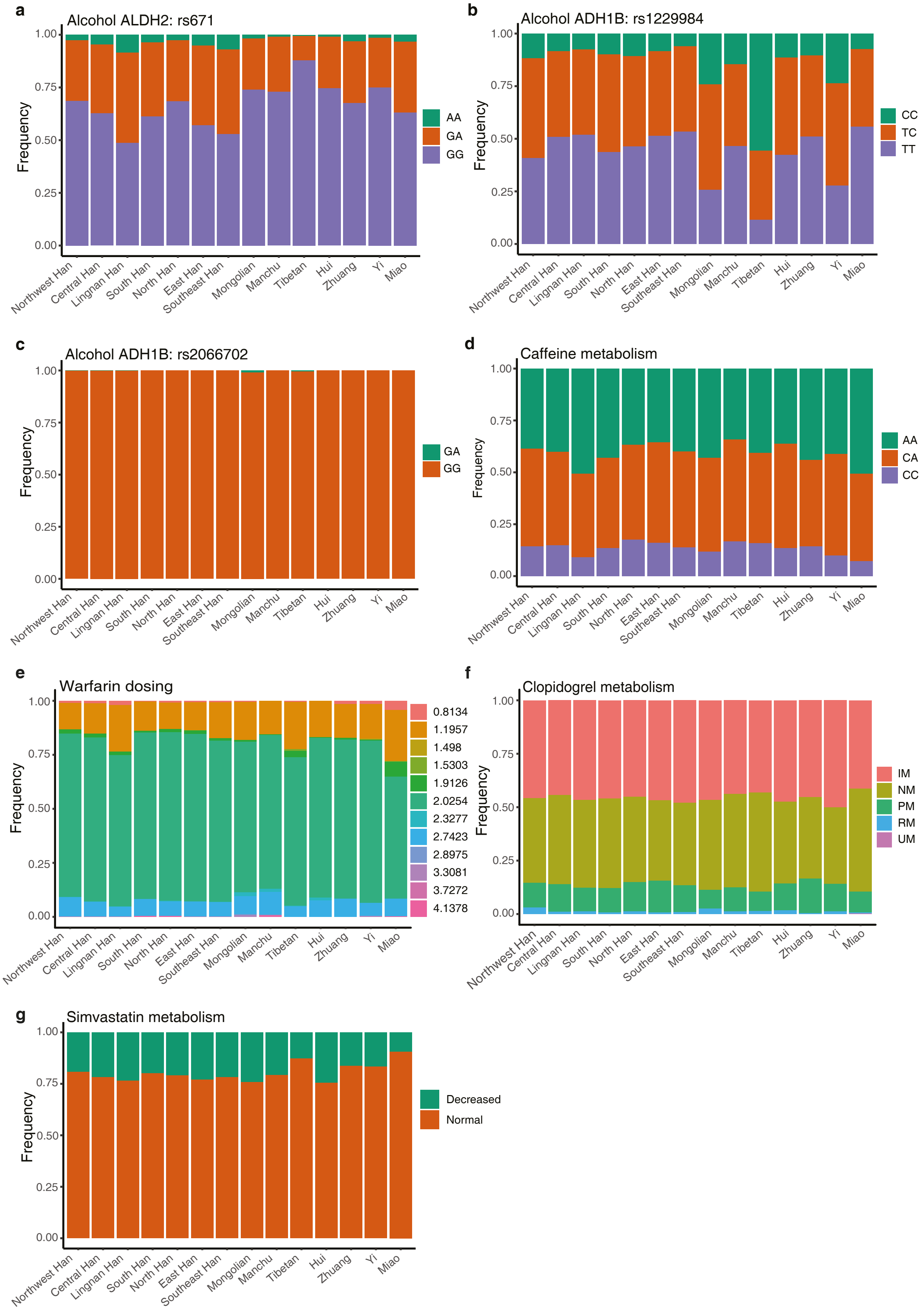
a
Alcohol ALDH2: rs671
Frequency
AA
GA
GG
b
Alcohol ADH1B: rs1229984
CC
TC
TT
c
Alcohol ADH1B: rs2066702
GA
GG
d
Caffeine metabolism
AA
CA
CC
e
Warfarin dosing
0.8134
1.1957
1.498
1.5303
1.9126
2.0254
2.3277
2.7423
2.8975
3.3081
3.7272
4.1378
f
Clopidogrel metabolism
IM
NM
PM
RM
UM
g
Simvastatin metabolism
Decreased
Normal
0.00
0.25
0.50
0.75
1.00
Northwest Han
Central Han
Lingnan Han
South Han
North Han
East Han
Southeast Han
Mongolian
Manchu
Tibetan
Hui
Zhuang
Yi
Miao

Fig. S7 The prevalence and distribution of critical variants for nutrition and drug metabolism. a-d The allele frequencies of critical variants associated with alcohol and caffeine metabolism in different Chinese populations. **e** The distribution of warfarin dosing in different Chinese populations. **f** The distribution of clopidogrel metabolism in different Chinese populations. NM, normal metabolizer; IM, intermediate metabolizer; UM, ultrarapid metabolizer; RM, rapid metabolizer; PM, poor metabolizer. **g** The distribution of simvastatin metabolism (normal and decreased capacity) in different Chinese populations.
